# Riociguate: Uma Alternativa para Tratar a Hipertensão Pulmonar

**DOI:** 10.36660/abc.20220305

**Published:** 2022-07-07

**Authors:** Gisele Zapata-Sudo

**Affiliations:** 1 Universidade Federal do Rio de Janeiro Faculdade de Medicina Instituto de Ciências Biomédicas Rio de Janeiro RJ Brasil Instituto de Ciências Biomédicas e Instituto do Coração Edson Saad, Faculdade de Medicina, Universidade Federal do Rio de Janeiro, Rio de Janeiro, RJ – Brasil

**Keywords:** Hipertensão Pulmonar/terapia, Hipertensão Pulmonar/fisiopatologia, Ativadores de Enzimas/uso terapêutico, Riociguat/uso terapêutico, Pirazóis/uso terapêutico, Pirimidinas/uso terapêutico

Nos últimos anos, avanços significativos foram alcançados no conhecimento da patologia da hipertensão pulmonar (HP), o que foi realizado em um esforço de pesquisa para identificar novas estratégias de tratamento. Entre os 5 subgrupos clínicos da HP, a mais comum é a hipertensão arterial pulmonar (HAP) idiopática, associada ao aumento da morbidade e mortalidade.^1^ Capacidade de exercício, classe funcional da OMS, valores hemodinâmicos, achados de imagem e biomarcadores de disfunção miocárdica são parâmetros utilizados para predizer a sobrevida de pacientes com HP.^2^ Este é um grande desafio clínico; a melhora da qualidade de vida dos pacientes e a variabilidade entre as terapias pioram porque se espera uma decisão adequada do profissional de saúde, pois pode afetar o resultado. O diagnóstico rápido é essencial e pode justificar que todos os pacientes com diagnósticos suspeitos sejam encaminhados a um centro especializado. O diagnóstico rápido é essencial e pode justificar que todos os pacientes com diagnósticos suspeitos sejam encaminhados a um centro especializado. O tratamento depende da classificação da HP, incluindo principalmente as drogas específicas isoladas ou em combinação que têm como alvo os inibidores da fosfodiesterase tipo 5 (PDE5i),^3^ estimuladores solúveis da guanilato ciclase (GCs), antagonistas do receptor de endotelina, análogos de prostaciclina e agonistas do receptor de prostaciclina que interferem na disfunção vascular das artérias pulmonares.^4^ Como a HAP é uma doença que inclui vasoconstrição de arteríolas pré-capilares e lesões obstrutivas, hiperproliferativas e vasculares, essas drogas não têm como alvo o remodelamento vascular e certamente não melhoram a função cardíaca. Assim, é essencial a busca de vasodilatadores pulmonares que interfiram nestas relevantes vias moleculares.^5^

Na edição dos Arquivos Brasileiros de Cardiologia, Spilimbergo et al.,^6^ relatam um estudo de acompanhamento no qual pacientes com HP foram tratados com um estimulante do GCs, riociguate, que é aprovado para o tratamento da HAP porque aumenta a via do GMP cíclico do óxido nítrico (NO). Os autores descrevem a evolução dos casos vivos ao longo de 3 anos, com foco na HAP (tipo 1) e HP tromboembólica crônica (HPTEC, tipo 4). O riociguate aumenta a atividade do GCs, que é o receptor intracelular do NO, que tem efeitos vasodilatadores e antiproliferativos nos vasos sanguíneos, incluindo as artérias pulmonares. Considerando a coorte de 31 pacientes, 32% estavam em classe funcional II da OMS e esse valor aumentou para 71% após 3 anos de tratamento com riociguate. Os autores destacam que o riociguate interferiu no processo da doença porque a maioria dos pacientes tratados com riociguate demonstrou parâmetros de risco estáveis ou melhores em 3 anos de seguimento. Anteriormente, Ghofrani et al.,^7^ demonstraram que o riociguate, por meio da ativação direta do GCs, promoveu aumento do GMP cíclico e consequentemente vasodilatação pulmonar, e sua administração 3 vezes ao dia em pacientes com HAP melhorou as concentrações séricas de peptídeo natriurético tipo N terminal pro B (NT-proBNP), tempo para piora clínica e classe funcional da OMS.^7^ A redução dos níveis de NT-proBNP não foi observada por Spilimbergo et al.,^6^ possivelmente explicada pelo pequeno número de pacientes incluídos no estudo. Da mesma forma, em 2015, o estudo CHEST-2 descreveu que a administração prolongada de riociguate em pacientes com HPTEC melhorou o exercício e a capacidade funcional.^8,9^ Todas as classes de agentes específicos para HP são caras e não proporcionam a cura, mas reduzem a internação hospitalar e melhoram a capacidade funcional. O riociguate pode ser uma opção alternativa para pacientes com HAP que não respondem ao tratamento com PDE5i9, pois pode estimular GCs independentemente do NO.^10^

Há fortes evidências que sugerem que o riociguate é uma intervenção promissora para melhorar o prognóstico de pacientes com HP.
