# Supplementary material for: Riociguate: Uma Alternativa para Tratar a Hipertensão Pulmonar
Source: Arq Bras Cardiol. 2022 Jul 7;119(1):111–2. [Article in Portuguese] doi: 10.36660/abc.20220305 (PMC9352137; doi:10.36660/abc.20220305)
Supplement: Translation

# Translation: Riociguat: An Alternative to Treat Pulmonary Hypertension

**Authors:** Gisele Zapata-Sudo

**Affiliations:** 1 Rio de Janeiro RJ Brazil Instituto de Ciências Biomédicas and Instituto do Coração Edson Saad, Faculdade de Medicina, Universidade Federal do Rio de Janeiro, Rio de Janeiro, RJ – Brazil

**Keywords:** Hypertension Pulmonary/therapy, Hypertension Pulmonary/physiopathology, Enzyme Activators/therapeutic use, Riociguat/therapeutic use, Pyrazoles/therapeutic use, Pyrimidines/therapeutic use

In recent years, significant progress has been achieved regarding the knowledge of the pathobiology of pulmonary hypertension (PH), which was conducted in a research effort to identify new treatment strategies. Among the 5 clinical subgroups of PH, the most common is idiopathic pulmonary arterial hypertension (PAH), associated with increased morbidity and mortality rate.^1^ Exercise capacity, WHO functional class, hemodynamic values, findings on imaging, and biomarkers of myocardial dysfunction are parameters used to predict the survival of patients with PH.^2^ This is a great clinical challenge; the improvement of patients’ quality of life and the variability between therapies worsens because a proper care provider decision is expected since it might affect the outcome. Rapid diagnosis is essential and could justify that all patients with suspected diagnoses should be referred to an expert center. Treatment depends on the classification of PH, including primarily the specific drugs alone or in combination which target the phosphodiesterase type 5 inhibitors (PDE5i),^3^ soluble guanylate cyclase (GCs) stimulators, endothelin receptor antagonists, prostacyclin analogs, and prostacyclin receptor agonists which interfere with the vascular dysfunction of pulmonary arteries.^4^ Since PAH is a disease that includes vasoconstriction of pre-capillary arterioles and obstructive, hyperproliferative and vascular lesions, these drugs do not target vascular remodeling and certainly do not improve cardiac function. Thus, it is essential to search for pulmonary vasodilators that interfere with this relevant molecular pathways.^5^

In issue of the *Arquivos Brasileiros de Cardiologia*, Spilimbergo et al.^6^ report a follow-up study in which patients with PH were treated with an GCs stimulant, riociguat, which is approved for treating PAH because it augments the nitric oxide (NO)-cyclic GMP pathway. The authors describe live cases outcome spanning 3 years, focusing on PAH (type 1) and chronic thromboembolic PH (CTEPH, type 4). Riociguat increases the activity of GCs, which is the intracellular receptor for NO, that has vasodilatory and antiproliferative effects on blood vessels, including the pulmonary arteries. Considering the cohort of 31 patients, 32% were in WHO functional class II and this value increased to 71% after 3 years of treatment with riociguat. The authors highlighted that riociguat interfered with the disease process because most patients treated with riociguat demonstrated stable or better risk parameters at 3 years of follow-up. Previously, Ghofrani et al.^7^ demonstrated that riociguat, through the direct activation of GCs, promoted an increase in cyclic GMP and consequently pulmonary vasodilation, and its administration 3 times daily in patients with PAH improved serum N terminal pro B type natriuretic peptide (NT-proBNP) concentrations, time to clinical worsening, and WHO functional class.^7^ Reduction of NT-proBNP levels was not observed by Spilimbergo et al.,^6^ possibly explained by the small number of patients included in the study. Similarly, in 2015, the CHEST-2 study described that long-term administration of riociguat in patients with CTEPH improved exercise and functional capacity.^8,9^ All classes of PH-specific agents are expensive and will not provide the cure but reduce hospital admission and improve functional capacity. Riociguat might be an alternative option for patients with PAH who do not respond sufficiently to treatment with PDE5i^9^ since it can stimulate GCs independently of NO.^10^

There is strong evidence to suggest that riociguat is a promising intervention to improve the prognosis of patients with PH.
